# ARHGEF3 Associated with Invasion, Metastasis, and Proliferation in Human Osteosarcoma

**DOI:** 10.1155/2021/3381957

**Published:** 2021-07-24

**Authors:** Jie Gong, Wei Tang, Bin Lv, Shushu Zhang, Tingjuan Fan, Guangyu Gao, Dong Chen, Yulong Liu

**Affiliations:** ^1^Department of Orthopedics, Suzhou Xiangcheng People's Hospital, 1060 Huayuan Road, Suzhou 215004, China; ^2^Department of Ultrasound, The Second Affiliated Hospital of Soochow University, Suzhou 215004, China; ^3^Jiangxi Key Laboratory of Cancer Metastasis and Precision Treatment, The Third Affiliated Hospital of Nanchang University, Nanchang, Jiangxi, China; ^4^Department of Oncology, The Second Affiliated Hospital of Soochow University, 1055, Sanxiang Road, Suzhou, 215004 Jiangsu, China; ^5^State Key Laboratory of Radiation Medicine and Protection, School of Radiation Medicine and Protection, Soochow University, Suzhou 215123, China; ^6^Collaborative Innovation Center of Radiological Medicine of Jiangsu Higher Education Institutions, Suzhou 215123, China

## Abstract

**Background:**

Osteosarcoma is a malignant bone tumor composed of mesenchymal cells producing osteoid and immature bone. This study is aimed at developing novel potential prognostic biomarkers and constructing a miRNA-mRNA network for progression in osteosarcoma.

**Method:**

GSE70367 and GSE70414 were obtained in the Gene Expression Omnibus (GEO) database. GEO software and the GEO2R calculation method were used to analyze two gene profiles. The coexpression of differentially expressed miRNAs (DEMs) and genes (DEGs) was identified and searched for in the FunRich database for pathway and ontology analysis. Cytoscape was utilized to construct the mRNA-miRNA network. Survival analysis of identified miRNAs and mRNAs was performed by utilizing the Kaplan-Meier Plotter. Besides, expression levels of DEMs and target mRNAs were verified by performing quantitative real-time PCR (qRT-PCR) and Western blot (WB).

**Results:**

Six differentially expressed microRNAs (DEMs) were identified, and 8 target genes were selected after screening. By using the KM Plotter software, miRNA-124 and ARHGEF3 were obviously associated with the overall survival of patients with osteosarcoma. Furthermore, ARHGEF3 was found downregulated in osteosarcoma cells by performing qRT-PCR and WB experiments. Results also showed that downregulated ARHGEF3 may associate with invasion, metastasis, and proliferation.

**Conclusions:**

By using microarray and bioinformatics analysis, DEMs were selected, and a complete miRNA-mRNA network was constructed. ARHGEF3 may act as a therapeutic and prognostic target of osteosarcoma.

## 1. Background

Osteosarcoma (OS) is the most common primary malignant carcinoma in bone tissues, which is characterized by malignant osteogenesis and malignant osteoblast differentiation [1]. Because of its rapid progress and poor prognosis, it is the main lethal disease in adolescence. Due to the lack of effective tumor early diagnostic markers, despite the rapid development of surgery, chemotherapy, radiotherapy, and other treatment methods, the long-term survival rate of patients with metastatic diseases remains at 25-30% [2]. The prognosis of advanced patients is still worse. Its etiology is not clear, but its occurrence and development may be regulated by genetic factors [3]. Therefore, it is urgent to have a deep understanding of its basic biology to determine its prognostic biomarkers and therapeutic targets [4].

MicroRNA (miRNA) is a class of noncoding single-stranded RNA molecules with a length of about 22 nucleotides encoded by endogenous genes. They are involved in the regulation of post transcriptional gene expression in animals and plants [5, 6]. For example, Liu and Cui found that miRNA-98-5p inhibits the progression of osteosarcoma by regulating cell cycle via targeting cell division cycle 25 expression [7]. Cai et al. found that overexpression of microRNA-206 accelerates osteosarcoma cells to proliferate and metastasize by targeting Notch3 [8]. Xu et al. also reported that downregulated microRNA-184 may reduce the cancer size of OS through regulation of the Wnt/*β*-catenin signaling pathway and may suffer a new method for the diagnosis and treatment of OS [9]. These findings suggest that the abnormal expression of miRNAs may be related to the occurrence and development of cancer, which may be through regulating cancer-related genes and participating in the pathogenesis of OS.

In our research, we identified differentially expressed genes and microRNAs by analyzing 1 osteosarcoma mRNA microarray dataset and 1 microRNA dataset. Our objective to identify the key genes in OS with survival, mRNA-microRNA interaction, ontology enrichment, and network analyses.

## 2. Methods

### 2.1. Microarray Data

GEO database is a database for storing chip, second-generation sequencing, and other high-throughput sequencing data. Using this database, we can retrieve some experimental sequencing data uploaded by others. In this article, GSE70367 and GSE70414 were achieved from GEO.

GSE70367, including 5 osteosarcoma cell lines and 1 human mesenchymal stem cell line (hMSC).. miRNA expression profiling research of these cell lines was conducted on an Affymetrix Multispecies miRNA-3 Array (GPL16384). Dataset GSE70414, 5 OS cell lines and 1 hMSC, was offered by the Affymetrix Human Genome U133 Plus 2.0 Array (GPL570).

### 2.2. Differently Expressed miRNA Analysis

GEO2R (https://www.ncbi.nlm.nih.gov/geo/geo2r/) is an interactive web tool, which permits people to compare two or more sets of samples in the GEO Series to select DEGs under different experimental situations. The results showed that it was a gene table in order of importance. GEO2R uses the GEOquery and limma R packages in the Bioconductor project to compare the processed tables provided by the original committer. In this study, GEO2R was utilized to select the DEMs and DEGs between the OS and human mesenchymal stem cells. Furthermore, ∣log2FC | ≥3 and *P* < 0.05 were utilized as a cut-off criterion, and an obvious statistical difference would be thought if the statistics were up to our standards.

### 2.3. Functional and Pathway Enrichment Analysis

FunRich (http://www.funrich.org) is an independent software tool, mainly used for functional enrichment and interaction network analysis of genes and proteins. The analysis results can be drawn into different types of pictures. Users can not only search the default background database but also load the custom database, which can be used for function-rich analysis. Cytoscape software was utilized to carry out KEGG (Kyoto Encyclopedia of Genes and Genomes) enrichment analysis. ClueGO is a data visualization tool based on the plug-in of Cytoscape. It supports the enrichment analysis of more than ten common databases such as GO, KEGG, Reactome, and miRBase. Finally, the enrichment results are displayed in the form of a network.

### 2.4. Prediction of Potential DEM Target mRNAs and MicroRNA-mRNA Regulatory Network

miRNAs inhibit the expression of target genes mainly by binding to target mRNA, promoting the degradation of mRNA, or hindering its translation. Accurate and rapid prediction of miRNA target genes by bioinformatics methods can provide clues for the study of miRNA function. Upregulated and downregulated DEMs were submitted to the FunRich online program to achieve target genes. Besides, GSE70414 was obtained from the GEO database. By combining the target genes from FunRich software and differential expression analysis of GSE70414, the intersection genes between the two results were selected and the microRNA-mRNA network was constructed by utilizing Cytoscape.

### 2.5. Analysis of the MicroRNAs and their Association with Osteosarcoma Prognosis

The Kaplan-Meier Plotter (http://www.kmplot.com/) is an online website for survival analysis. At present, the website can carry out research on 54,675 genes and 18,674 cancer samples, involving breast cancer, lung cancer, etc.; the data types include chip data and high-throughput sequencing data, involving mRNA and miRNA, and are constantly enriched [11]. In this study, we divided OS patients into two groups (high expression and low expression) according to the expression of specific genes. The overall survival of OS patients was analyzed by the KM Plotter database.

### 2.6. Cell Culture and Antibodies

The human OS cell lines (MG63, 143B, U2OS, U2R) were offered by Professor Kang Tie Bang (Sun Yat-sen University Cancer Center, State Key Laboratory of Oncology in South China, Collaborative Innovation Center for Cancer Medicine, Guangzhou, China). Osteosarcoma cells and hFOB1.19 cells were cultured in Dulbecco's modified Eagle's medium (DMEM; Thermo Fisher Scientific) supplemented with 10% fetal bovine serum (FBS; Gibco) in an incubator. The incubator is at 37 degrees Celsius with 5% CO_2_. Anti-*β*-actin and anti-ARHGEF3 (rabbit) antibodies were purchased from the Proteintech Group, Inc. (Wuhan, China).

### 2.7. Quantitative Real-Time PCR

Total RNA was extracted from OS cell lines and hFOB1.19 cells using TRIzol reagents (Invitrogen, Carlsbad, CA, USA) according to the manufacturer's instructions. The total RNA extracted was amplified by RT-PCR using SYBR Green Supermix (Bio-Rad Laboratories) according to the manufacturer's instructions, and mRNA levels of ARHGEF3 were detected. The results were calculated by the 2^−*ΔΔ*Ct^ method. We found ARHGEF3 primer pairs in previous studies, and the primer pairs are shown in Table 1. The reference group was GAPDH to avoid errors caused by factors such as sampling error, RNA quantitative error, and nonuniform amplification efficiency of each PCR system.

### 2.8. Protein Extraction and Western Blotting

Osteosarcoma cells and hFOB1.19 cells were collected by centrifugation and then washed with PBS. The resulting precipitate was added to the radio immunoprecipitation assay (RIPA) buffer (Thermo Fisher Scientific) and then cracked on ice for half an hour. The cracked sample was centrifuged in a 4°C centrifuge at 12,000 rpm for 20 minutes, and the supernatant was left. Bradford assay (Bio-Rad Laboratories) was used to quantify the protein. Western blot assays were performed on the total protein obtained. The protein was separated on 10% sodium dodecyl sulfate-polyacrylamide gel (SDS-PAGE) and then transferred to a polyvinylidene fluoride (PVDF) membrane (Millipore, USA). The resulting PVDF membrane was placed in 5% skimmed milk and blocked for about 1 hour. The specific antibody was then incubated overnight at 4°C, washed three times with PBST (1 × PBS with Tween) the next day, and then incubated at room temperature for 1 hour with the corresponding secondary antibody (rabbit). The strips were incubated with the secondary antibody and washed three times with PBST, and finally, autoradiography was performed with an ECL kit (Thermo Fisher Scientific).

### 2.9. Cell Transfection

The oe-ARHGEF3 sequence was cloned into pLKO.1 vector, and the stable system with overexpression ARHGEF3 was constructed for the next experiment. ARHGEF3 oeRNA were purchased from GenePharma Co. Ltd. (Suzhou, China). Lipofectamine 2000 and Lipofectamine® RNAiMAX were used as the transfection reagents according to the manufacturer's instructions.

### 2.10. Transwell Assays

Transwell chambers were placed on a 24-well plate, and osteosarcoma cells in the logarithmic growth phase were digested with trypsin and washed with PBS and serum-free medium. 1∗10^5^ cells and serum-free medium were inoculated in the upper compartment of each well. DMEM medium containing 10% fetal bovine serum was added to the lower compartment. For cell invasion assay, 50 *μ*l diluted matrix glue (BD Biosciences, Franklin Lakes, NJ) was added to the upper chamber and incubated at 37°C for 4-5 hours; the following operation is the same as above. The cells were cultured in a 37°C incubator for 22 hours in the migration experiment and 24 hours in the invasion experiment. The cells that have migrated or invaded are fixed, stained, and counted at a specified time.

### 2.11. Wound Healing Assays

Wound healing assays were constructed to measure cell migration and repair. We used a 20 UL pipette tip to line the central growth area of adherent cells cultured on a 12-well plate. The floating cells in the central part were washed with PBS, and the cells were cultured for another 24 hours. Besides, we observed and filmed the migration process 0 and 36 hours after scratch. After that, we also measured and calculated the distance between the two edges of the scratch.

## 3. Results

### 3.1. Identification of the DEGs between Osteosarcoma Cell Lines and Human Mesenchymal Stem Cells

**Figure 1:**
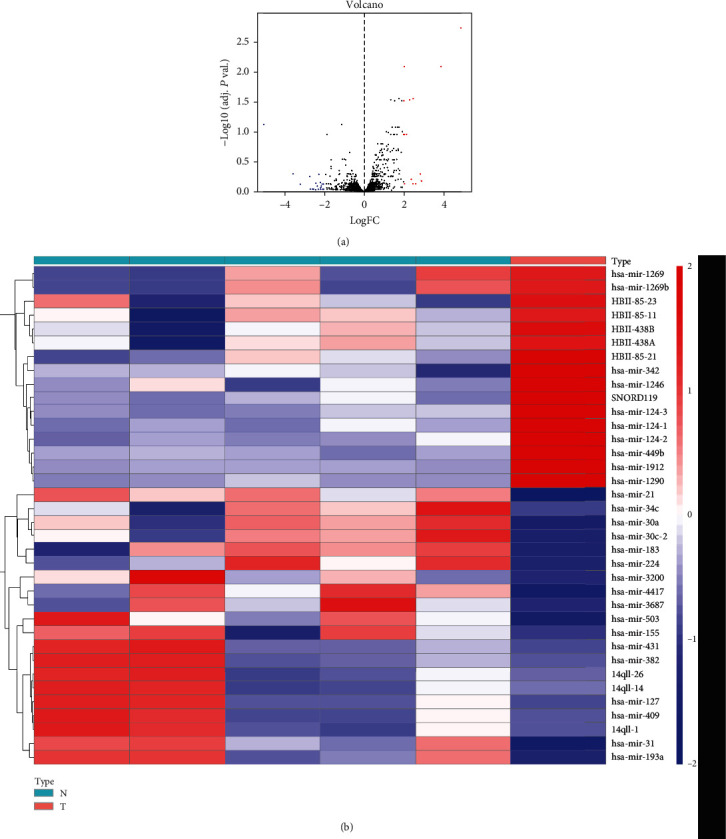
Heat map and volcano map of differentially expressed genes of GSE48074.

**Figure 2:**
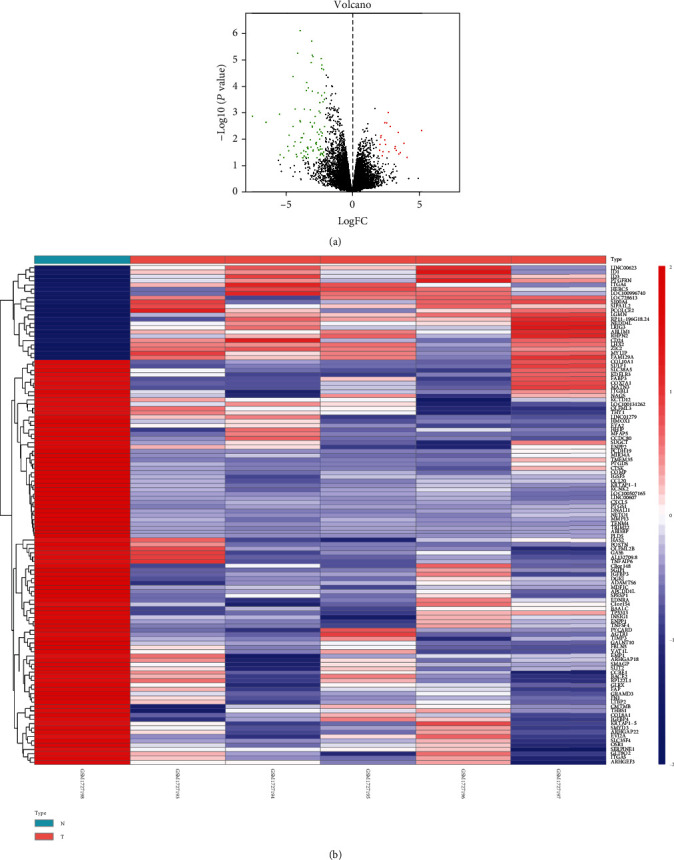
Heat map and volcano map of differentially expressed genes of GSE70574.

GEO2R was utilized to analyze the miRNA and mRNA expression profiles from the GSE70367 and GSE70414. Based on the cut-off criteria (*P* < 0.05 and ∣log2FC | ≥2), 36 DEMs including hsa-microRNA-1290, hsa-microRNA-124-3, hsa-microRNA-1912, hsa-microRNA-224, hsa-microRNA-31, and hsa-microRNA-21 (Figure 1) and 118 differentially expressed mRNAs were identified (Figure 2).

### 3.2. Screening of Potential Transcription Factors and Enrichment Analysis

**Figure 3:**
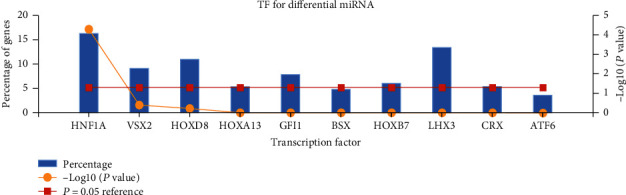
Identification of the potential transcription factors of DEMs by FunRich software.

**Figure 4:**
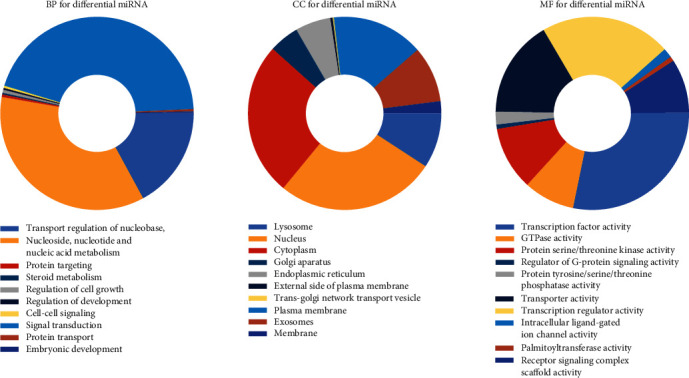
The top 10 of the biological process, cellular component, and molecular function of the target genes of miRNAs.

**Figure 5:**
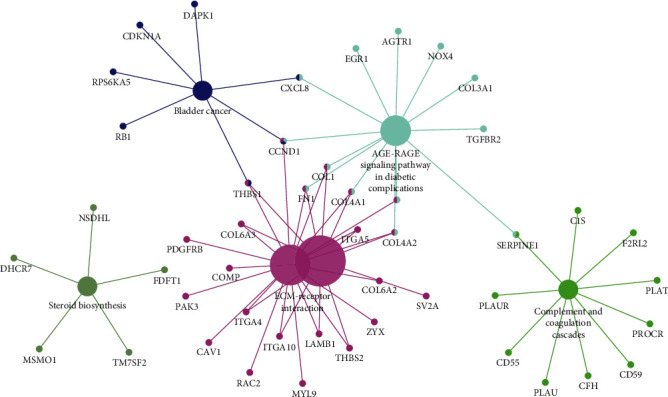
KEGG pathway enrichment analysis of potential target mRNAs.

Transcription factor enrichment research found HNF1A, VSX2, HOXD8, HOXA13, GFI1, BSX, HOXB7, LHX3, CRX, and ATF6 (Figure 3). To further understand the function of the selected miRNAs, FunRich was utilized to carry out GO enrichment research. The result indicated that DE-microRNAs were most enriched in the lysosome, nucleus, cytoplasm, transcription factor activity, GTPase activity, protein serine/threonine kinase activity, transport, protein targeting, and regulation of nucleobase, nucleoside, nucleotide, and nucleic acid metabolism (Figure 4). We also utilized Cytoscape and ClueGO to conduct the KEGG pathway analysis. These identified microRNAs were mainly enriched in 6 pathways including glycosphingolipid biosynthesis, retinol metabolism, AGE-RAGE signaling pathway in diabetic complications, pancreatic secretion, inflammatory mediator regulation of TRP channels, and renin secretion (Figure 5).

### 3.3. miRNA-mRNA Regulatory Network

**Figure 6:**
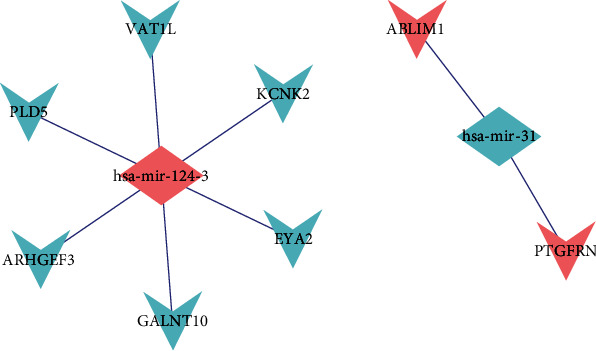
Identified target mRNAs and miRNA-mRNA regulatory network.

By utilizing FunRich software, 1610 potential target mRNAs were obtained, and only 8 of them differentially expressed in GSE70414 (ARHGEF3, EYA2, GALNT10, KCNK2, PLD5, VAT1L, ABLIM1, and PTGFRN). To show the composition and relationship of miRNA and mRNA more clearly, we built a microRNA-mRNA network by utilizing Cytoscape software. Finally, 8 essential microRNA-mRNA pairs (microRNA-124-3 and microRNA-31) were selected which implied the vital effect of osteosarcoma (Figure 6).

### 3.4. Research of the MicroRNAs and Their Association with Osteosarcoma Prognosis

**Figure 7:**
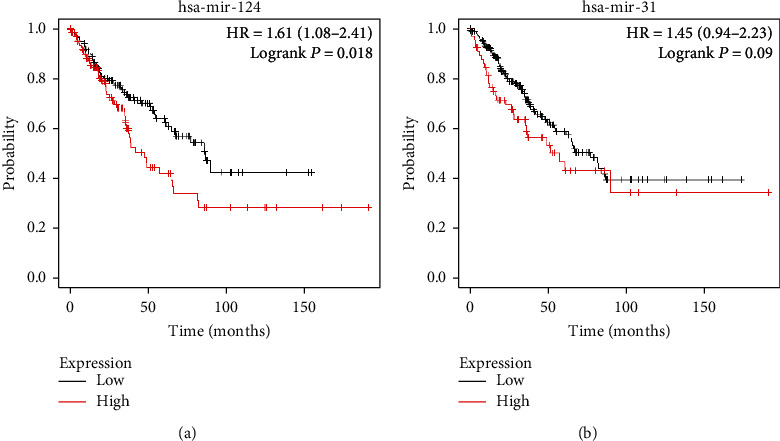
The association between the expression level of selected miRNAs and osteosarcoma prognosis.

**Figure 8:**
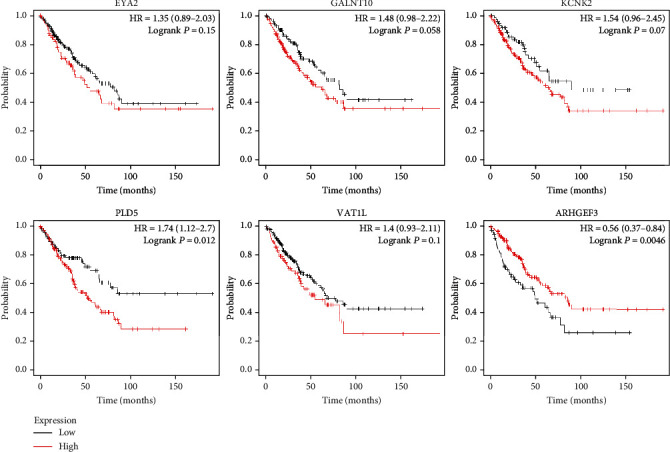
The association between the expression level of target mRNAs and osteosarcoma prognosis.

KM Plotter was used to research the survival of patients with osteosarcoma. By uploading 2 microRNAs we identified, we achieved 2 survival curves. The outcome demonstrated that microRNA-124 (Figure 7) were associated with prognosis of patients with OS. However, the expression of microRNA-31 may have no obvious association with the survival rate. Next, hsa-mir-124 was chosen for further study. By uploading 6 target mRNAs, the corresponding survival curve was achieved (Figure 8). The outcome indicated that the expression level of ARHGEF3 and PLD5 may have an obvious association with the overall survival of patients with osteosarcoma. This indicated that the identified microRNAs and mRNAs may be potential targets.

### 3.5. Validation of the Expression with qRT-PCR and Western Blot

**Figure 9:**
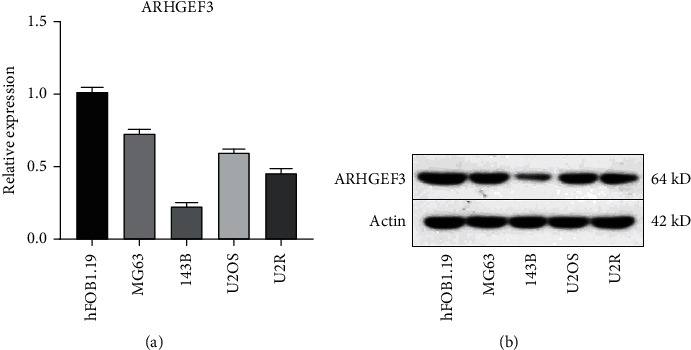
(a) The mRNA expression level of ARHGEF3 in various OS cell lines and osteoblastic cell lines was determined by qRT-PCR. Data are presented as mean ± SD. ^∗^*P* < 0.05 vs. hFOB1.19. (b) The expression level of ARHGRF3 in hFOB1.19 and osteosarcoma cell lines (detected by Western blot).

To further assess the expression of ARHGEF3, we selected four osteosarcoma cell lines to detect its mRNA level and protein expression, and the control group was the normal osteoblast hFOB1.19. Consistent with the microarray data, ARHGEF3 was highly expressed in hFOB1.19 cells and was low expressed in various osteosarcoma cell lines at both mRNA and protein levels (Figures 9(a) and 9(b)). Although the difference in the expression of other cell lines was not as significant as that of 143B cells, this result can prove to some extent that the high expression of ARHGEF3 in osteosarcoma cell lines may be associated with poor prognosis of osteosarcoma cells.

### 3.6. Overexpression of ARHGEF3 Inhibited the Aggressiveness of 143B Cells in the Osteosarcoma Cell Line

**Figure 10:**
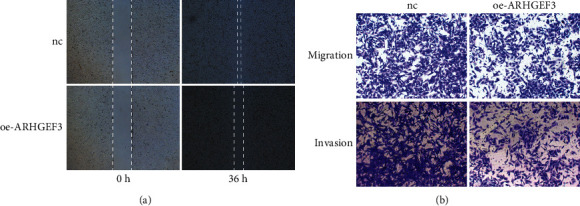
(a) Wound healing assays in the overexpression cell line and NC. (b) The migration and invasion assays. Measurements were in triplicate, and data were presented as the mean ± SD. ^∗∗^*P* < 0.01 vs. hFOB1.19 (scale: 10x).

**Figure 11:**
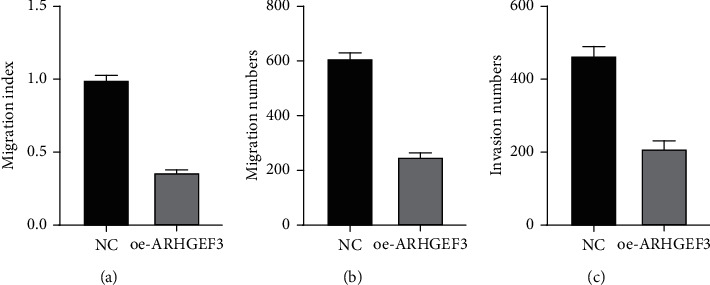
(a) Migration index of wound healing assays in the overexpression cell line and NC. (b) The migration numbers. (c) The invasion numbers. Measurements were in triplicate, and data were presented as the mean ± SD. ^∗∗^*P* < 0.01 vs. hFOB1.19.

According to the expression level results obtained in the above experiments, we selected the cell line 143B with the lowest expression level for the next experiment. To determine the biological significance of ARHGEF3, we transfected 143B cells with oeRNA (oe-ARHGEF3) to upregulate ARHGEF3 gene expression, and the oeRNA was introduced into 143B cells by the lentiviral vector to establish stable cell lines (143B/oe-ARHGEF3 and 143B/NC). Previous studies on ARHGEF3 have found that the low expression of ARHGEF3 promotes the invasion and repair of many tumors. Therefore, to know whether ARHGEF3 has the same performance in osteosarcoma, we conducted migration, invasion, and wound healing assays. In wound healing assays, results showed that the healing rate of 143B/oe-ARHGEF3 stable cells was significantly slower than that of 143B/NC stable cells at 36 h. In the transwell experiment, we can see that compared with 143B/NC cells, 143B/oe-ARHGEF3 stable cells migrated and invaded the lower compartment in a significantly reduced number. These results indicated that overexpression ARHGEF3 could inhibit the migration, invasion, and healing of 143B cells and that high ARHGEF3 expression promoted the invasiveness of 143B cells in an osteosarcoma cell line (Figures 10 and 11).

## 4. Discussion

At present, much evidence shows that miRNAs and mRNAs are the key regulators of tumorigenesis, development, spread, and inhibition of tumor cell proliferation or induction of apoptosis. At the same time, a gene-targeting therapy strategy should also be considered, which is related to inhibiting the growth and metastasis of osteosarcoma [10, 11]. In our study, GSE70367 and GSE70414 were obtained in the GEO database. 6 DE-microRNAs including microRNA-1290, microRNA-124-3, microRNA-1912, microRNA-224, microRNA-31, and microRNA-21 were selected. To further learn the function of the 6 microRNAs in osteosarcoma, FunRich was utilized for further study. Transcription factor enrichment analysis demonstrated that these microRNAs were primarily associated with the HNF1A, VSX2, HOXD8, HOXA13, GFI1, BSX, HOXB7, LHX3, CRX, and ATF6. Gene Ontology enrichment analysis indicated that these miRNAs were associated with lysosome, nucleus, cytoplasm, transcription factor activity, GTPase activity, protein serine/threonine kinase activity, transport, protein targeting, and regulation of nucleobase, nucleoside, nucleotide, and nucleic acid metabolism. This is consistent with the recognition that lysosomes and the nucleus play an important role in some common human diseases [12]. As for GTPase activity, it is a key component of many intracellular processes. The incorrect regulation of G protein is related to diseases: cancer [13–15], cardiovascular disease [16], genetic disease [17], and so on. Kyoto Encyclopedia of Genes and Genomes pathway analysis demonstrated that these identified microRNAs were mainly enriched in 6 pathways. The pathways of focal adhesion in mankind have been researched for several years. It plays an oncogenic role in cancer invasiveness and metastasis. Besides, previous research has proven the relationship between focal adhesion activation and metastasis in breast cancer [18]. Inhabiting focal adhesion could slow metastasis formation of mammary cancers [19, 20]. Therefore, it has been identified as a potential therapeutic target for aggressive breast carcinoma [21]. As for the ECM-receptor interaction pathway, Zhang et al. reported that Twist-related protein 2 may promote migration and invasion of kidney cancer cells by regulating ITGA6 and CD44 expression associated with the ECM-receptor-interaction pathway [22]. It may also involve the development of breast carcinoma [23]. However, the function of these pathways in osteosarcoma has not been described in detail yet.

The microRNA-mRNA network was constructed on the basis of FunRich and Cytoscape. Six microRNAs were identified for further study. Besides, 1610 potential target mRNAs were obtained and only 8 of them differentially expressed in GSE70414 (ARHGEF3, EYA2, GALNT10, KCNK2, PLD5, VAT1L, ABLIM1, and PTGFRN). Besides, some microRNAs have been confirmed to be associated with cancer biological behavior. For example, a previous research indicated that microRNA-1290 promoted proliferation, sphere formation, colony formation, and invasion of NSCLC [24]. It took part in the radio resistance of human cervical tumor cells and chemoresistance in hepatocellular cancer [25]. Besides, another study concluded that microRNA-1290 sensitized the Wnt pathway and added the reprogramming-related transcript factors c-Myc and Nanog in colorectal carcinoma [26]. As for hsa-mir-124-3, it is a subtype of miR-124 and highly expressed in the mammalian nervous system. Besides, it might act as a potential target for the diagnosis and treatment of hepatocellular carcinoma [27]. It was also found to improve GATA6 expression and be related to a poor prognosis in cholangiocarcinoma [28]. In osteosarcoma, it was found that the expression of miR-124 was significantly downregulated in tumor patients when compared to periostitis patients and healthy controls. The conclusion indicated that microRNA-124 was an independent prognostic indicator for OS [29]. As for hsa-mir-31, Tu et al. reported that it was induced by ultraviolet which was correlated with chronic actinic dermatitis severity, which acted as a vital role in regulating the keratinocyte permeability barrier through targeting CLDN1 [30]. It looks like a novel metastatic colorectal cancer marker whose expression level permits for the selection of patients with wild-type KRAS cancer who more possibly respond to anti-EGFR treatment [31]. In chordoma, it was found differentially expressed compared with healthy nucleus pulposus and may serve as a prognostic factor and therapeutic target [32]. By using FunRich software, 1610 potential target genes were obtained and only 8 of them differentially expressed in GSE70414 (ARHGEF3, EYA2, GALNT10, KCNK2, PLD5, VAT1L, ABLIM1, and PTGFRN). As a result, a microRNA-mRNA network was built including 2 microRNAs (microRNA-124-3 and microRNA-31) and 8 mRNAs.

ARHGEF3 (Rho Guanine Nucleotide Exchange Factor 3) is a protein coding gene. ARHGEF3-related diseases include methotrexate-related lymphoproliferation and osteoporosis. A previous study reported that overexpression of ARHGEF3 played a vital oncogenic role in nasopharyngeal carcinoma pathogenesis by preventing cell apoptosis through the overexpression of baculoviral IAP repeat containing 8, and ARHGEF3 might be used as a new prognosis biomarker and effective therapeutic target for human NPC [33]. It also sensitizes acute myeloid leukemia differentiation via activation of RhoA signaling pathways directly regulated by small GTPase family proteins [34]. Besides, it may act as potential regulators of several mRNAs in bone cells, including TNFRSF11B, ARHGDIA, PTH1R, and ACTA2, which heavily affects osteoblast-like and osteoclast-like cells [35].

GALNT10 (Polypeptide N-Acetylgalactosaminyltransferase 10) is an enzyme that mediates the modification of proteins and lipids. It plays an important role in normal cell progression and other physiological processes. It is also a vital marker for the reason that can offer particular goals for therapeutic interventions [36]. A previous research indicated that overexpression of GALNT10 is associated with an immunosuppressive microenvironment to accelerate cancer development and predicts worse clinical results in high grade ovarian serous carcinoma patients [36]. Besides, it could also promote gastric cancer cell metastasis ability and reduce the sensitivity to 5-fluorouracil by regulating Homeobox D13 [37].

EYA2 (EYA Transcriptional Coactivator and Phosphatase 2) is a protein coding gene. Diseases related to EYA2 include syndromic microphthalmia and malignant peripheral nerve sheath tumor. A previous study reported that it can promote the invasion of human astrocytoma according to the regulation of extracellular signal-regulated kinase/matrix metalloproteinase signaling [38]. Besides, it can promote cell proliferation, invasion, and tumor progression by regulating docetaxel sensitivity and mitochondrial membrane potential, possibly through the AKT/Bcl-2 axis [39]. In lung adenocarcinoma, the expression of EYA2 was found upregulated in cancer tissues and may be involved in the progression of lung adenocarcinoma as a transcriptional activator [40].

Survival analysis demonstrated that expression of microRNA-124-3 was related to worse prognosis of patients with osteosarcoma by using KM Plotter software. Similarly, after uploading 8 miRNAs, we got the corresponding survival curves. The results indicated that upregulated ARHGEF3 and PLD5 were associated with poor overall survival. PLD5 is a protein that in humans is encoded by the phospholipase D family, member 5 genes. According to previous studies, PLD was found upregulated in many tumor cells such as triple-negative breast cancer [41], ovarian cancer [42], and colorectal cancer [43]. However, as for PLD5, according to searching the relevant literature, we did not find any evidence that it was related to the tumor. Survival analysis also indicated that hsa-mir-124 and target mRNAs (ARHGEF3 and PLD5) may have an obvious association with the prognosis of patients with osteosarcoma.

Since miRNAs generally negatively regulate target mRNAs, we chose ARHGEF3 for further study according to the survival curves. The WB and qRT-PCR results revealed that the expression level of ARHGEF3 in osteosarcoma (MG63, 143B, U2OS, and U2R) was indeed much lower than that in human osteoblasts (hFOB1.19), especially in the 143B cell line. Therefore, we selected 143B cells to conduct the ARHGEF3 overexpression experiment and conducted Transwell assays and wound healing assays on the cells that had been overexpressed with ARHGEF3, and the results showed that ARHGEF3 overexpression does reduce the invasion and healing ability of 143B cells. In conclusion, we can infer that ARHGEF3 acts as a biomarker in OS as in other tumors.

Nowadays, with the development of precision medicine, the individual differences of treatment methods are paid more and more attention. Therefore, it is important to find novel markers and treatment ways. Our research demonstrated that microRNA-124 and ARHGEF3 were involved in the development of OS by some signaling pathways and had prognostic worth. Since our primary analysis was achieved from the GEO database by using R software, more experiments are needed to verify our conclusions and explore the mechanism.

## 5. Conclusion

Our research demonstrated certain mechanisms for the progression of osteosarcoma. Also, has-miR-124 and ARHGEF3 were selected as potential biomarkers of OS. However, these results need more research to prove them.

## Figures and Tables

**Table 1:** The primers for quantitative real-time polymerase chain reaction with GAPDH used as the internal standard control.

	Forward	Reverse
ARHGEF3	5′-GCCAGGATCGATATGGTTGCGAAGGACTAC-3′	5′-AAGCTAGAATTCGCTCTCTCACAGGGCTGAC-3′
GAPDH	5′-TGGTATCGTGGAAGGACTCATGAC-3	5′-ATGCCAGTGAGCTTCCCGTTCAGC-3

## Data Availability

The datasets used and/or analyzed during the current study are available from the corresponding author on reasonable request.
